# Adherence to national guidelines for the diagnosis and management of severe malaria: a nationwide, cross-sectional survey in Malawi, 2012

**DOI:** 10.1186/s12936-016-1423-2

**Published:** 2016-07-19

**Authors:** Monica P. Shah, Melissa Briggs-Hagen, Jobiba Chinkhumba, Andy Bauleni, Alfred Chalira, Dubulao Moyo, Wilfred Dodoli, Misheck Luhanga, John Sande, Doreen Ali, Julie Gutman, Don P. Mathanga, Kim A. Lindblade

**Affiliations:** Malaria Branch, Division of Parasitic Diseases and Malaria, Centers for Disease Control and Prevention, 1600 Clifton Road NE, Mailstop A-06, Atlanta, GA 30333 USA; Malaria Alert Centre, Malawi College of Medicine, Blantyre, Malawi; National Malaria Control Programme, Malawi Ministry of Health, Lilongwe, Malawi; World Health Organization, Lilongwe, Malawi

**Keywords:** Severe malaria, Case management, Diagnosis, Treatment, Malawi

## Abstract

**Background:**

Severe malaria has a case fatality rate of 10-20 %; however, few studies have addressed the quality of severe malaria case management. This study evaluated the diagnostic and treatment practices of malaria patients admitted to inpatient health facilities (HF) in Malawi.

**Methods:**

In July–August 2012, a nationwide, cross-sectional survey of severe malaria management was conducted in 36 HFs selected with equal probability from all eligible public sector HFs in Malawi. Patient records from all admissions during October 2011 and April 2012 (low and high season, respectively) were screened for an admission diagnosis of malaria or prescription of any anti-malarial. Eligible records were stratified by age (< 5 or ≥ 5 years). A maximum of eight records was randomly selected within each age and month stratum. Severe malaria was defined by admission diagnosis or documentation of at least one sign or symptom of severe malaria. Treatment with intravenous (IV) quinine or artesunate was considered correct. Patients without documentation of severe malaria were analysed as uncomplicated malaria patients; treatment with an artemisinin-based combination therapy (ACT) or oral quinine based on malaria test results was considered correct. All analyses accounted for HF level clustering and sampling weights.

**Results:**

The analysis included 906 records from 35 HFs. Among these, 42 % (95 % confidence interval [CI] 35–49) had a severe malaria admission diagnosis and 50 % (95 % CI 44–57) had at least one severe malaria sign or symptom documented. Severe malaria patients defined by admission diagnosis (93, 95 % CI 86–99) were more likely to be treated correctly compared to patients defined by a severe sign (82, 95 % CI 75–89) (p < 0.0001). Among uncomplicated malaria patients, 26 % (95 % CI 18–35) were correctly treated and 53 % (95 % CI 42–64) were adequately treated with IV quinine alone or in combination with an ACT or oral quinine.

**Conclusions:**

A majority of patients diagnosed with severe malaria received the recommended IV therapy in accordance with national treatment guidelines. However, the inconsistencies between diagnosis of severe malaria and documentation of severe signs and symptoms highlight the need to improve healthcare worker recognition and documentation of severe signs and symptoms.

**Electronic supplementary material:**

The online version of this article (doi:10.1186/s12936-016-1423-2) contains supplementary material, which is available to authorized users.

## Background

Despite the availability of effective interventions for the prevention and treatment of uncomplicated malaria, the burden of severe malaria remains considerable. In 2014, malaria was responsible for an estimated 438,000 deaths worldwide, with a majority of deaths occurring in sub-Saharan Africa and in children under 5 years of age [1]. Young children in areas of stable transmission are at increased risk of progression to severe disease compared to adults [2] and more frequently present with impaired consciousness, respiratory distress, multiple convulsions, severe anaemia, hypoglycaemia, acidosis, hyperlactataemia, and hyperparasitaemia [2–4]. Among hospitalized patients under 5 years of age, the case fatality rate for severe malaria is estimated to be between 10–20 % [4–6]. Prompt care is essential as most deaths occur within the first 24 h of hospital admission [7].

Optimal case management of severe malaria is complex and requires prompt recognition of clinical manifestations of severe malaria, initiation of appropriate treatment, monitoring of disease progression, and management of co-morbidities. These processes also rely on the availability of health system resources such as diagnostic and treatment supplies. Given that effective case management of severe malaria is multi-faceted, few studies have sought to evaluate the quality of patient care in sub-Saharan Africa. In Uganda, a 2009 health facility survey of severe malaria case management practices found that, of all patients assessed, only 27 % were correctly diagnosed with severe malaria and 30 % did not receive the correct initial parenteral anti-malarial at the appropriate dose and frequency. Although 54 % of facilities reported no stock-outs of the recommended parenteral quinine in the 3 months prior to the survey, no facilities had consistent availability of all supplies required for the management of severe malaria [8].

Malaria is a leading cause of hospital admissions and hospital deaths in Malawi and places considerable stress on the health system. In 2010, malaria was responsible for 40 % of all hospital admissions in children under 5 years of age, 34 % of all outpatient visits, and an estimated 40 % of all hospital deaths [9]. These challenges and the burden of severe malaria in Malawi underscore the need to assess severe malaria case management practices.

The objective of this survey was to better understand how severe malaria is currently managed at public inpatient health facilities in Malawi. Specifically, the quality of care given to severe malaria patients admitted to inpatient health facilities with respect to the 2011 Malawi Revised Guide for the Management of Malaria [10] was evaluated.

## Methods

### Study setting

Malaria is endemic and occurs year-round in Malawi, with peak transmission during the rainy season from November to April. Malaria infections are predominantly caused by *Plasmodium falciparum* and the national parasite prevalence among children under 5 years of age in 2012 was estimated to be 28 % by microscopy and 43 % by rapid diagnostic test (RDT) [11].

In Malawi, primary care services including the management of uncomplicated malaria are available at health centers, community hospitals, and district hospitals. Secondary care services, including management of severe malaria, are provided at community or district hospitals. Publicly funded health facilities are managed by either the government, which provides care at no cost, or the Christian Health Association of Malawi (CHAM), which requires a small fee for services [12].

According to the Malawi Revised Guide for the Management of Malaria, released in 2011, severe malaria is diagnosed based on the presence of one or more severe clinical or laboratory findings. Although parasite confirmation by microscopy is recommended, parenteral treatment with either quinine or artesunate should be initiated immediately if severe malaria is suspected. For uncomplicated malaria, the guidelines recommend laboratory confirmation by RDT or microscopy prior to treatment initiation, and administration of artemether-lumefantrine (AL) as first-line or artesunate-amodiaquine as second-line treatment for confirmed cases.

### Study design

In July–August 2012, a nationwide, cross-sectional survey of inpatient malaria case management was conducted in 36 health facilities systematically selected with equal probability from a list of all public sector health facilities in Malawi that admit patients with malaria. Health facilities were ordered by region (north, central, and south), managing authority (government and CHAM) and hospital type (district, community, or other) prior to systematic random selection. In the selected facilities, survey teams conducted interviews of patients admitted for severe malaria, interviews of healthcare workers to assess knowledge, training, and supervision, an evaluation of health facility supplies and capacity, and retrospective reviews of health records of previously admitted patients. The methodology and results from the retrospective reviews of patient health records are described here.

### Sampling of patient records and sample size determination

Records from all patients admitted to medical wards, pediatric wards or intensive care units during October 2011 (low malaria season) and April 2012 (high malaria season) were screened for an admission diagnosis of malaria or prescription of any anti-malarial drug. Eligible records meeting these criteria were then stratified by age (<5 and ≥5 years). A maximum of eight patient records was randomly selected within each age group and month stratum for a total of up to 32 records per health facility.

The sample size was estimated based on a conservative outcome proportion of 50 %, design effect of 1.8, precision of ±10 %, and type I error of 5 %, resulting in 269 records per stratum or 1076 in total. After adjustment for a potential loss of 10 % due to missing records, the final required sample size was determined to be eight patient records per stratum.

### Data management and analysis

Patient records were abstracted by medically trained surveyors on a standardized form. The abstracted forms were electronically entered independently by two data entry clerks and all discrepancies were resolved by consulting the data abstraction forms. The two definitions of severe malaria described below were analysed as separate outcomes. Estimations of proportions and 95 % confidence intervals (CIs) were calculated using survey procedures in SAS version 9.3 (SAS Institute Inc., Cary, NC) to account for clustering at the health facility level and weighted by the inverse of the probability of selection. Results were stratified by age (<5 and ≥5 years), region, health facility managing authority, and month of admission. Rao–Scott Chi Square was used to test for differences between categorical variables. Risk ratios (RRs) were estimated using a log-binomial regression model with a generalized estimating equations approach to account for correlation from repeated measures within the same health facility. The populations of severe malaria patients defined based on admission diagnosis and the population defined based on documentation of a severe sign were not independent and, therefore, comparisons between the proportions of severe malaria patients correctly treated by each definition were made by accounting for repeated measures at the patient level. For all statistical tests, a p < 0.05 was considered significant.

### Definitions

#### Diagnosis

Severe malaria was defined and analysed in two ways:

1. By admission diagnosis of severe malaria, cerebral malaria, or severe anaemia as documented by the healthcare worker.
2. By documentation of at least one sign of severe malaria, including any of the following: seizures, lethargy, coma, vomiting everything, unable to eat, jaundice, difficulty breathing, difficulty urinating, red urine, easy bleeding/bruising, abnormal mental status, abnormal neurologic exam, or severe anaemia (haemoglobin level <5 grams per decilitre).

Patients who were admitted for malaria or who received an anti-malarial, and did not meet either of the above criteria for severe malaria were considered to have suspected or confirmed (by microscopy or RDT) uncomplicated malaria.

#### Treatment

*Severe malaria*

- Correct treatment was defined as treatment with either intravenous (IV) quinine or IV artesunate.
- Under-treatment was defined as treatment with no or ineffective anti-malarial treatment.

*Uncomplicated malaria*

**Fig. 1:**
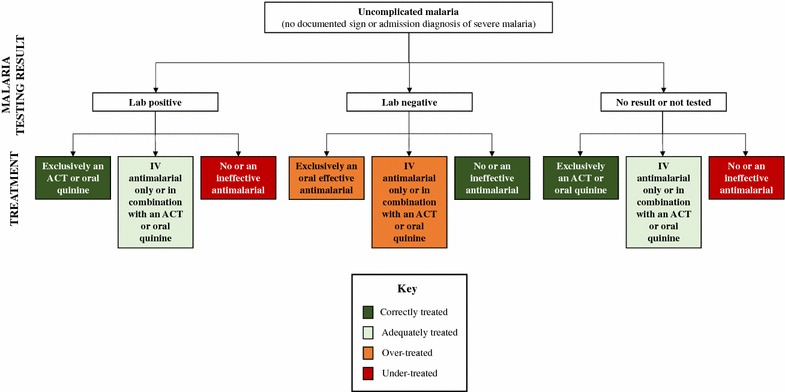
Treatment definitions for uncomplicated malaria patients. *ACT* artemisinin-based combination therapy; *IV* intravenous
- Adequate treatment was defined as treatment with an IV anti-malarial (considered clinically effective, but not in line with recommended national guidelines) among laboratory-confirmed or clinically diagnosed uncomplicated malaria patients (Fig. 1).
- Under-treatment was defined as no or ineffective anti-malarial treatment among laboratory-confirmed or clinically diagnosed uncomplicated malaria patients (Fig. 1).
- Over-treatment was defined as treatment with an IV anti-malarial, ACT, or oral quinine among patients without documentation of a sign or admission diagnosis of severe malaria with a negative laboratory result (Fig. 1).

- Correct treatment was defined as treatment exclusively with an artemisinin-based combination therapy (ACT) or oral quinine among laboratory-confirmed or clinically diagnosed uncomplicated malaria patients (Fig. 1).

## Results

**Fig. 2:**
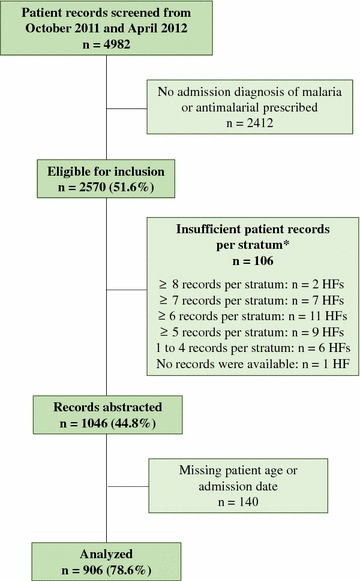
Study profile of patient records screened and analysed. *HF* health facility. (*Asterisk*) Desired sample size was 1076 patient records. After adjustment for potential loss of 10 % due to missing data, the sample size was equivalent to 8 patient records per age and month strata. Most HFs had an insufficient number of records per strata; missing records were not replaced

Among the 36 health facilities surveyed, 4982 patient records were screened for inclusion from October 2011 and April 2012. Fever was documented in 53 % of all patient records, of which, 77 % were eligible for inclusion. Approximately half (52 %) of patient records screened had an admission diagnosis of malaria or prescription of an anti-malarial. No records were available at one of the sampled health facilities and only two facilities had sufficient eligible patient records in all four strata to reach the target sample size. Of the remaining facilities, seven had at least seven records per stratum, 11 had at least six records per stratum, nine had at least five records per stratum, and six had between one and four records per stratum. At facilities with an insufficient number of patient records, all eligible records were abstracted and analysed. A total of 906 patient records with complete data across 35 health facilities were included in this analysis (Fig. 2).

### Characteristics of patients analysed

Patients were between 3 months and 81 years of age and approximately half (52 %) were female. Although most patients were febrile at the time of admission, patients <5 years were more likely to present with fever (94 %) than patients ≥5 years (79 %) (p < 0.0001). Laboratory testing for malaria was performed for 67 % of patients overall (70 % in children <5 years vs. 61 % in patients ≥5 years, p = 0.04) and microscopy was performed in 56 % of all patients tested for malaria, with no significant difference by age. Laboratory-confirmed malaria was significantly higher in children <5 years compared to patients ≥5 years (53 vs. 37 %, respectively; p < 0.0001) and in patients admitted in April 2012 (high malaria season) compared to October 2011 (low season) (55 vs. 35 %, respectively; p < 0.0001). A majority of patients (>90 %) were discharged alive and well, while the remaining patients were either discharged with morbidity or died during admission (Table 1).

**Table 1:** Characteristics of suspected malaria patients by age, region, health facility managing authority, and month of admission

Characteristic	Age in years, n (%)			Region, n (%)				Managing authority, n (%)			Admission month, n (%)		
	<5	≥5	p value	North	Central	South	p value	Govt.	CHAM	p value	October 2011	April 2012	p value
All patients	*463 (58)*	*443 (42)*	*0.0003*	218 (30)	368 (38)	320 (32)	0.87	447 (43)	459 (58)	0.41	*453 (44)*	*453 (56)*	<*0.0001*
Female	240 (52)	258 (58)	0.19	114 (52)	203 (55)	181 (56)	0.63	237 (53)	261 (56)	0.46	251 (53)	247 (56)	0.47
Pregnant	0 (0)	28 (6)	–	3 (1)	14 (3)	11 (3)	0.28	13 (2)	15 (2)	0.84	*24 (5)*	*4 (1)*	<*0.0001*
Febrile	*427 (94)*	*335 (79)*	<*0.0001*	185 (89)	311 (87)	266 (87)	0.84	376 (87)	386 (88)	0.61	371 (85)	391 (90)	0.075
*Malaria testing*													
RDT or microscopy performed	*320 (70)*	*290 (61)*	*0.040*	135 (56)	222 (64)	253 (78)	0.20	254 (57)	356 (72)	0.17	292 (61)	318 (70)	0.052
Positive result	*235 (53)*	*172 (37)*	<*0.0001*	79 (34)	170 (50)	161 (54)	0.13	174 (41)	233 (50)	0.19	*162 (35)*	*245 (55)*	<*0.0001*
*Disposition*													
Alive and well	431 (96)	400 (96)	0.99	201 (97)	332 (95)	298 (97)	0.79	402 (95)	429 (97)	0.24	408 (95)	423 (97)	0.13
Alive with morbidity	9 (2)	12 (2)		4 (1)	13 (2)	4 (1)		14 (2)	7 (1)		14 (2)	7 (1)	
Died	12 (2)	13 (2)		6 (3)	9 (2)	10 (2)		16 (3)	9 (2)		15 (3)	10 (2)	

All p values determined by Rao-Scott Chi Square. Italicized results reflect p < 0.05.

*Govt*. Government; *CHAM* Christian Health Association of Malawi; *RDT* Rapid Diagnostic Test

### Diagnosis of severe malaria

The most common admission diagnosis was unspecified malaria (51 %), followed by severe malaria (40 %). Uncomplicated malaria was diagnosed in 2 % of patients, severe anaemia was diagnosed in 2 % and cerebral malaria was diagnosed in <1 % (Table 2). Overall, 42 % (95 % confidence interval [CI] 35–49) of patients had a severe malaria admission diagnosis (including diagnosis of severe malaria, cerebral malaria, or severe anaemia). An admission diagnosis of severe malaria was significantly more likely in April 2012 (high season, 48 %) than in October 2011 (low season, 35 %) (p = 0.0006), in government facilities (52 %) than in CHAM facilities (35 %) (p = 0.0008) and in children <5 years (48 %) compared to patients ≥5 years (34 %) (p = 0.002). No significant differences in admission diagnoses were observed by region.

**Table 2:** Health Worker diagnosis at admission, by age

Admission Diagnosis	<5 years		≥5 years		p value
	n	% (95% CI)	n	% (95% CI)	
Severe malaria*	213	45.4 (37.8–52.9)	153	32.1 (24.0–40.3)	0.0040
Cerebral malaria	1	0.3 (0.0–0.8)	3	0.4 (0.0–1.0)	–
Uncomplicated malaria	10	1.5 (0.4–2.7)	14	2.9 (0.8–5.1)	0.089
Malaria (unspecified)*	209	46.3 (38.2–54.5)	240	57.8 (48.0–67.6)	0.011
Severe anaemia	11	2.2 (0.5–3.9)	6	1.1 (0.2–1.9)	0.079
Sepsis	3	0.4 (0.0–1.1)	2	0.3 (0.0–0.7)	–
Pneumonia	4	0.7 (0.0–1.5)	1	0.3 (0.0–1.0)	–
Gastroenteritis/Dehydration	1	0.3 (0.0–0.8)	3	0.5 (0.0–1.6)	–
Other	6	1.5 (0.0–3.3)	15	3.4 (1.3–5.5)	0.13
Not documented	5	1.5 (0.1–2.9)	6	1.3 (0.1–2.4)	0.82
Severe malaria by study definition*^,^ **	225	47.8 (40.2–55.4)	162	33.6 (25.1–42.1)	0.0021

p value determined by Rao–Scott Chi Square

* p < 0.05

** Includes admission diagnosis of severe malaria, cerebral malaria, or severe anemia as documented by the health worker

Half (50, 95 % CI 44–57) of patients had at least one severe malaria sign or symptom documented. Overall, the most commonly documented severe symptoms were seizures (20 %), lethargy/coma (15 %), difficulty breathing (9 %), and severe anaemia (7 %). Severe anaemia was higher among patients admitted in government facilities (10 %) compared to CHAM facilities (5 %) (p = 0.04) and in children <5 years (10 %) compared to patients ≥5 years (4 %) (p = 0.0004). In addition, in children <5 years, seizures (24 %), jaundice (3 %), and difficulty breathing (12 %) were significantly more common than in patients ≥5 years (p < 0.03) (Table 3). However, children <5 years had a similar risk for any severe sign/symptom as patients ≥5 years (RR = 1.3, 95 % CI 1.0–1.8) and no significant differences by region, health facility, and month of admission were observed.

**Table 3:** Documented clinical or laboratory symptoms of severe malaria, by age

Symptom	<5 years		≥5 years		p value
	n	% (95% CI)	n	% (95% CI)	
Seizures*	118	23.8 (18.5–29.2)	51	14.2 (7.3–21.1)	0.026
Lethargy/Coma	74	15.1 (10.7–19.5)	65	15.5 (10.4–20.6)	0.91
Vomiting everything	24	4.4 (2.1–6.8)	17	3.1 (1.4–4.9)	0.39
Unable to eat*	38	7.4 (4.6–10.3)	19	5.0 (2.2–7.7)	0.18
Jaundice*	19	3.3 (1.0–5.6)	4	0.7 (0.0–1.6)	0.0059
Difficulty breathing*	61	12.3 (8.0–16.5)	24	4.4 (2.1–6.6)	0.0002
Difficulty urinating	6	1.1 (0.0–2.4)	5	1.1 (0.1–2.2)	0.98
Red urine	3	0.5 (0.0–1.2)	2	0.6 (0.0–1.5)	–
Easy bleeding/bruising	0	0	0	0	–
Abnormal mental status	14	2.5 (1.1–3.9)	22	4.3 (1.7–6.9)	0.19
Abnormal neurologic exam	11	2.2 (1.0–3.4)	13	2.8 (1.2–4.3)	0.56
Severe anaemia*	51	9.6 (4.9–14.2)	20	3.8 (1.8–5.7)	0.0004
At least one severe symptom	287	56.1 (47.8–64.3)	177	42.4 (30.8–54.1)	0.06

p value determined by Rao–Scott Chi square

* p < 0.05

**Fig. 3:**
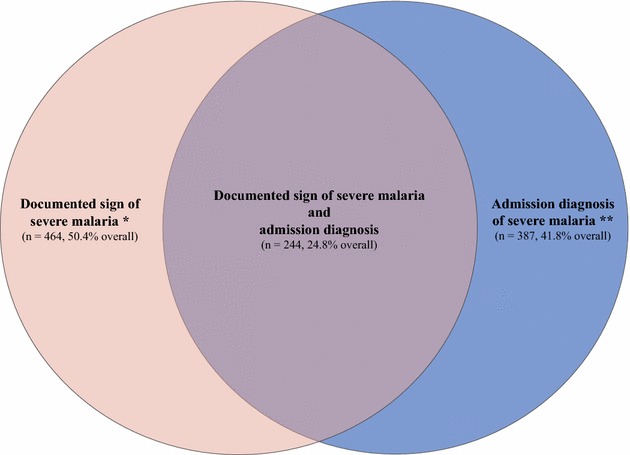
Patient distribution by definition of severe malaria. More than half of patients (59 %) with an admission diagnosis of severe malaria (shaded in *blue*) also had a documented sign of severe disease, while 49 % with a documented sign (shaded in *red*) were diagnosed with severe malaria at admission. Overall, 25 % of patients had severe malaria both by admission diagnosis and by documentation of a severe sign (shaded in *purple*). (*Asterisk*) At least one documented clinical or laboratory sign of severe malaria. (*Double* *asterisk*) Admission diagnosis of severe malaria, cerebral malaria, or severe anemia as documented by the healthcare worker

Of the 40 % of patients admitted with a diagnosis of severe malaria, 59 % also had a documented sign of severe disease (shown in blue, Fig. 3). In addition, almost half (44 %) of patients without an admission diagnosis of severe malaria had documentation of a severe sign. Thus, only 49 % of those with a documented sign of severe disease were diagnosed with severe malaria at admission (shown in red, Fig. 3). Overall, 25 % of patients had severe malaria both by admission diagnosis and by documentation of a severe sign (shown in purple, Fig. 3). Given that approximately half of patients could be misclassified by using one definition alone or many patients would be misclassified by restricting to those meeting the criteria for both definitions, severe malaria diagnosis by documentation of severe sign and by admission diagnosis were analysed separately.

### Treatment of severe malaria patients

**Fig. 4:**
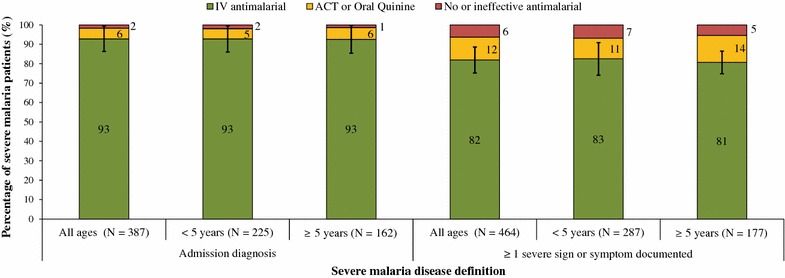
Treatment of severe malaria, by definition of severe disease and by age. *ACT* artemisinin-based combination therapy; *IV* intravenous. Error bars indicate 95 % confidence interval for the proportion correctly treated

Overall, 85 % (95 % CI 79–91) of all severe malaria patients, either defined by admission diagnosis or by sign of severe disease were treated correctly with recommended IV therapy. Among all ages, significantly more severe malaria patients by admission diagnosis (93, 95 % CI 86–99) were correctly treated compared to patients defined by severe sign (82, 95 % CI 75–89) (p < 0.0001). The proportion of severe malaria patients correctly treated did not differ significantly by age for either definition of severe disease. All severe malaria patients that were treated with an IV anti-malarial received quinine. The remaining severe malaria patients received either exclusively an ACT or oral quinine (6 or 12 %, by admission diagnosis or by severe sign, respectively) or did not receive any anti-malarial (2 or 6 %, by admission diagnosis or by severe sign, respectively) (Fig. 4).

### Diagnosis of uncomplicated malaria

Patients without documentation of a severe sign and without an admission diagnosis of severe malaria were analysed as suspected or confirmed uncomplicated malaria patients (N = 299). Among these patients, 119 (43 %) had a laboratory confirmed diagnosis, 80 (24 %) had a negative laboratory test for malaria, and 100 (33 %) were not tested or did not have a malaria test result documented (Additional file 1).

### Treatment of uncomplicated malaria patients

**Fig. 5:**
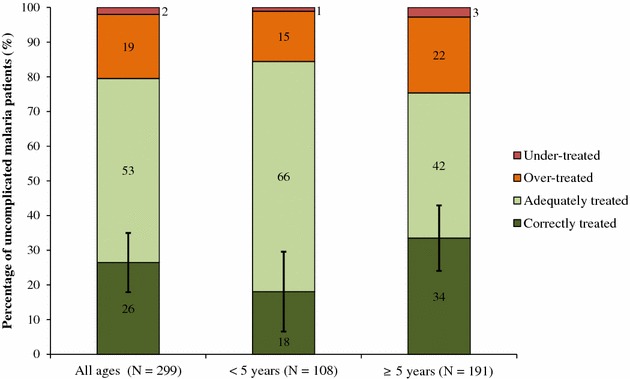
Treatment of uncomplicated malaria, by age. Error bars indicate 95 % confidence interval for the proportion correctly treated

Overall, 26 % (95 % CI 18–35) of uncomplicated malaria patients were correctly treated, either with an ACT or oral quinine exclusively (80 %) or no anti-malarial for patients with a negative malaria test result (20 %). Of the patients correctly treated with an oral anti-malarial exclusively, 81 % received AL, 18 % received oral quinine, and 2 % received both. No patients received artesunate-amodiaquine. Half of all uncomplicated malaria patients (53, 95 % CI 42–64) were adequately treated with IV quinine alone or in combination with an ACT or oral quinine. A notable proportion of patients (19, 95 % CI 12–25) were treated with one or more anti-malarials despite a negative test result (over-treated) and, among these patients, 53 % (95 % CI 41–66) received an IV anti-malarial. Few uncomplicated malaria patients (2, 95 % CI 0–4) were under-treated with an ineffective anti-malarial or no treatment (Fig. 5).

## Discussion

In this nationwide health facility survey assessing inpatient management of severe malaria in Malawi, malaria accounted for a high burden of hospital admissions (52 %) and most patients admitted with severe malaria were treated with the correct IV anti-malarial in accordance with national treatment guidelines (85 %). However, diagnostic practices for severe malaria and case management practices for uncomplicated malaria were not consistently in line with the recommendations described in the 2011 Malawi Revised Guide for the Management of Malaria. Health worker recognition and/or documentation of severe malaria signs was found to be sub-optimal. In addition, the overuse of IV anti-malarial drugs for the treatment of uncomplicated malaria, which is not consistent with national guidelines, was observed in over half of uncomplicated malaria patients. The quality of documentation of patient records may have affected the results of this survey; however, the findings presented from this study reflect areas that can be improved in order to provide optimal care to malaria patients who are hospitalized for their illness.

Patient records were used to retrospectively evaluate severe malaria case management in this survey. Inconsistencies between admission diagnosis and the corresponding documentation of severe signs were observed. A large proportion of patients (31 %) with an admission diagnosis of severe malaria did not have a documented sign of severe disease and, conversely, many patients (51 %) with a documented sign of severe disease were not diagnosed with severe malaria. Assuming that the patients with an admission diagnosis of severe malaria were accurately diagnosed, the 31 % who lacked documentation of any severe signs are most likely a result of poor documentation practice. Several potential reasons could explain the absence of an admission diagnosis of severe malaria in the 51 % of patients with a documented severe sign.

First, many of the patients had an admission diagnosis of unspecified malaria, which may reflect a lack of documentation that the illness was severe malaria. Second, it is possible that the health care worker did not recognize the severe sign as a sign of severe malaria and, as a result, did not make the corresponding diagnosis of severe malaria. Finally, the severe sign could have developed after admission and, therefore, the admission diagnosis was accurate. Given the challenges with documentation, in this study the definitions of severe malaria diagnosis were analysed separately by both admission diagnosis and by documentation of a sign of severe disease. A 2009 health facility study in Uganda also found poor documentation of severe malaria signs, with only 28 % of severe malaria patient records indicating at least one severe sign [8]. Structured admission record forms have been shown to improve documentation of signs and symptoms [13] and, at the time of this survey, standardized admission forms were being introduced in many facilities in Malawi. More widespread implementation and use of these forms could improve documentation of signs as well as facilitate diagnoses made by health workers.

In accordance with the Malawi Revised Guide for the Management of Malaria (2011), severe malaria patients should be treated with IV quinine or IV artesunate upon clinical presentation of severe signs, even prior to blood smear confirmation, and IV treatment should be continued for parasitologically-confirmed patients. Therefore, any patients suspected of having severe malaria either by admission diagnosis or by documentation of a severe sign should have received a recommended IV anti-malarial. A majority of patients in this study (93 and 82 % by admission diagnosis and severe sign, respectively) were correctly treated with an IV anti-malarial; a very small percent received no anti-malarial (2 % by admission diagnosis and 6 % by severe sign), while the remaining patients received exclusively oral therapy (ACT or quinine) (6 % by admission diagnosis and 12 % by severe sign). Although parasitologic confirmation was not required for administration of IV therapy, 51 % of severe malaria patients (defined by either admission diagnosis or presence of a severe sign) who received the recommended IV therapy were confirmed malaria cases, 16 % were negative and 34 % did not have a documented malaria testing result. For the patients with a negative test result who were given IV therapy, it is possible that laboratory results were available after IV therapy had commenced and IV therapy was ceased once the negative result was determined. Of note, no patients receiving IV therapy were given artesunate. IV artesunate was included as recommended therapy for severe malaria in the 2011 guidelines; however, training of health care workers in the use of artesunate was not completed until 2015 and artesunate was not readily available at health facilities during the time frame of this study (Peter Troell; personal communication).

The finding that significantly more severe malaria patients defined by admission diagnosis received correct treatment compared to patients defined by severe sign suggests that health workers failed to recognize that these severe signs indicate the need for IV therapy. This speculation is supported by poor recognition of signs of severe disease during a malaria-knowledge assessment of health workers interviewed at the time of the survey in which 43 % of health workers were not able to name at least three signs of severe malaria (Briggs–Hagen, M; unpublished data). In addition to poor health worker knowledge, the lack of availability of medical supplies or recommended IV anti-malarials could have contributed to incorrect treatment. According to a health facility assessment conducted at the time of the survey, 26 % of facilities reported a stock-out of all anti-malarial treatments for severe malaria within the prior 3 months (Briggs–Hagen, M; unpublished data). However, given that the treatment stock-outs are unrelated to the definitions of severe disease, the observed difference in the proportion of patients correctly treated by definition of severe disease suggests that one of the alternative explanations proposed is more plausible.

Although the focus of this study was to assess the quality of severe malaria case management, the diagnosis and treatment of uncomplicated malaria patients in inpatient settings was also evaluated. National guidelines recommend that all patients suspected of uncomplicated malaria receive parasitological confirmation by either microscopy or RDT prior to treatment. In this study, one-third of uncomplicated malaria patients were either not tested or did not have a malaria test result documented. Although presumptive diagnosis by health workers may account for a proportion of patients not tested, the inadequate availability of diagnostic supplies in Malawi pose a challenge to universal diagnostic testing. RDTs were introduced in Malawi in 2011, and the roll-out to all government and CHAM facilities continued until the end of 2012; therefore, at the time points evaluated in this survey, RDT use may not have been introduced in some facilities. At the time of the survey, RDT stock-outs in the prior 3 months were reported in 44 % of health facilities (Briggs–Hagen, M; unpublished data). In addition, only some facilities had the capacity for microscopic diagnosis, as this requires electricity, availability of microscopes and slides, and trained microscopists, which were not consistently present. Improved health worker practices and availability of malaria testing supplies are required to improve diagnosis of uncomplicated malaria.

Appropriateness of treatment for suspected or confirmed uncomplicated malaria was evaluated based on malaria testing result. Only 26 % of uncomplicated malaria patients were treated correctly, while overuse of IV quinine, although clinically effective, was observed in 53 % of uncomplicated malaria patients. Although IV treatment is recommended in patients that present with vomiting at admission, 61 % of uncomplicated malaria patients treated with IV quinine did not have documented vomiting at admission. In addition among patients who received an admission diagnosis of uncomplicated malaria by the health worker, 13 % were treated with IV quinine. The overuse of IV quinine represents an unnecessary cost to the health system, and may exacerbate shortages leading to lack of availability for treatment of severe malaria patients. In this study, uncomplicated malaria was defined as the absence of a documented severe sign and without an admission diagnosis of severe malaria. It is possible that this led to misclassification of some patients who were appropriately treated with IV therapy for a severe sign which was not documented. However, among the 19 % of patients who received an anti-malarial despite a negative malaria test result, 53 % received an IV anti-malarial, suggesting true over-use of IV anti-malarials. Health worker non-adherence to negative malaria test results has been observed in numerous studies [14–19]. Reasons for non-adherence to negative results include provider distrust in test accuracy, patient dissatisfaction with diagnosis, or inability to provide a differential diagnosis for febrile-illness [20]. Among uncomplicated malaria patients in this survey, few were under-treated. Approximately 23 % of febrile patients screened were ineligible for inclusion in this study and may have accounted for additional uncomplicated malaria patients that were undertreated.

This study had several limitations. In low resource settings, patient medical records are often incomplete and data are supplemented with other sources such as interviews with patients and/or providers, or direct observation of consultations [21]. Given the retrospective nature of reviewing patient records, it was not possible to verify information with providers or patients in real time to correct inconsistencies or obtain missing information prior to analysis. Additionally, missing documentation of information such as signs, diagnoses, medications, and laboratory results was considered as the absence of these findings from the patient’s medical history and could have potentially led to misclassification of diagnosis and treatment outcomes in this study. Although survey tools were designed to capture treatment dose and regimen, these variables could not be included as part of correct treatment assessment due to the lack of documentation in patient records. Survey tools were also designed to capture the initiation and completion time of case management activities. However, time was rarely documented and, therefore, it was not possible to determine the sequence of case management events such as whether malaria testing results were received before or after initiation of IV therapy. Furthermore, the inclusion criteria for this survey restricted the analysis to suspected malaria patients, which limited the ability to generalize the study findings for all inpatient admissions.

## Conclusions

Malaria accounts for a considerable burden of hospital admissions in Malawi. In this survey, a majority of patients diagnosed with severe malaria received the recommended IV therapy in accordance with national treatment guidelines. However, given the inconsistencies observed between health worker diagnosis of severe malaria and documentation of severe signs, improvements to health worker recognition of severe signs and documentation are necessary to improve case management practices. Efforts to encourage the use of diagnostic supplies and recommended treatment for uncomplicated malaria are necessary since insufficient testing and over-treatment of uncomplicated malaria was observed. Ongoing efforts to ensure universal access to appropriate diagnostic supplies and treatment, further training to improve health worker recognition of signs of severe malaria, and improvements to record keeping are recommended.

## Additional file

10.1186/s12936-016-1423-2 Treatment of uncomplicated malaria, by laboratory testing status and age. Error bars indicate 95 % confidence interval for the proportion correctly treated.
