# Supplementary figures and images for: Adherence to national guidelines for the diagnosis and management of severe malaria: a nationwide, cross-sectional survey in Malawi, 2012
Source: Malar J. 2016 Jul 19;15:369. doi: 10.1186/s12936-016-1423-2 (PMC4950799; doi:10.1186/s12936-016-1423-2)

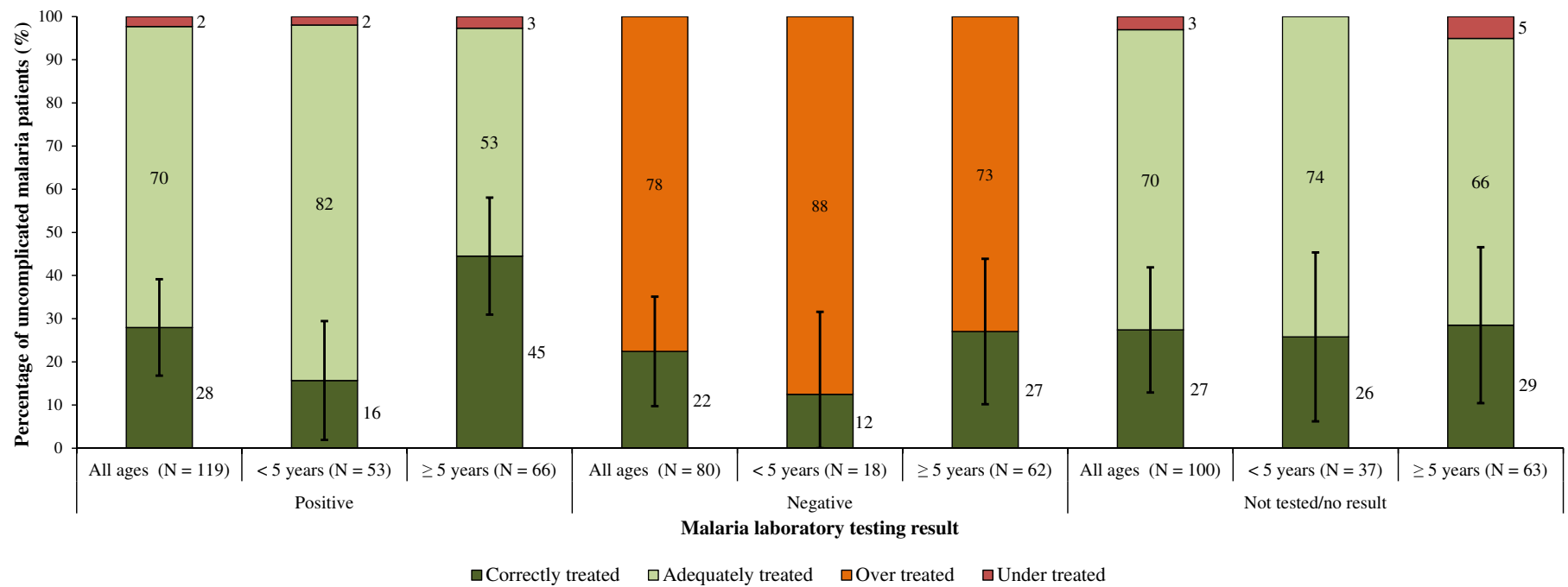

Supplement: Supplementary file 1 — 10.1186/s12936-016-1423-2 Treatment of uncomplicated malaria, by laboratory testing status and age. Error bars indicate 95 % confidence interval for the proportion correctly treated. [file 12936_2016_1423_MOESM1_ESM.pdf]
